# Tension Pneumocephalus From an Eroding Cholesteatoma: A Case Report and Review of the Literature

**DOI:** 10.7759/cureus.12873

**Published:** 2021-01-23

**Authors:** Muhammad Z Khan, Abdur Jamil, Danial Tahir, Ramsha Sidiq

**Affiliations:** 1 Internal Medicine, Central Michigan University College of Medicine, Saginaw, USA; 2 Internal Medicine, Central Michigan University, Saginaw, USA; 3 General Medicine/Pediatrics, Ayub Medical College, Abbottabad, PAK; 4 Internal Medicine, Quaid-e-Azam Medical College, Bahawalpur, PAK

**Keywords:** pneumocephalus, tension pneumocephalus, cholesteatoma, otogenic, hyperoxia, craniotomy

## Abstract

Pneumocephalus is defined as the presence of air inside the cranial vault. Benign and tension pneumocephalus are different ends of the same disease spectrum. Tension pneumocephalus leads to the formation of a pressure gradient, requiring emergent surgical decompression to prevent herniation of the intracranial structures. In this report, we present a rare case of tension pneumocephalus with essentially benign radiological findings secondary to a ruptured cholesteatoma. The patient was a 64-year-old woman with a history of end-stage renal disease on hemodialysis and hypertension. She presented to the emergency department (ED) with acute-onset weakness and decreased mentation. Physical exam findings were consistent with a cerebrovascular accident (CVA). CT scan and CT angiogram (CTA) were unremarkable for ischemia or hemorrhage but showed signs of free intracranial air, consistent with the diagnosis of pneumocephalus. After the activation of the code stroke, neurosurgery and neurology were consulted. Worsening respiratory status led to a decision to proceed with emergent intubation, but it was held based on the family’s decision to proceed with comfort measures. The patient’s status declined further within minutes and she died. Afterward, the case was discussed with the radiologist, who interpreted the cause as a cholesteatoma that had eroded through the temporal bone.

## Introduction

Pneumocephalus or the presence of air inside the cranium is often seen secondary to neurotrauma. Iatrogenic causes, neoplasms, and infections pertaining to the sinuses or middle ear cavity constitute about one-fourth of the cases seen. Cholesteatoma eroding into the cranium has not been frequently reported as a causative factor. Although a relatively benign condition seen after neurosurgeries, pneumocephalus at times can evolve into tension pneumocephalus, which carries significant mortality and is considered an emergency [1]. Less than 4% of middle ear pathologies lead to intracranial complications but the associated mortality turns out to be greater than 10% [2]. The distinction between uncomplicated and rapidly evolving tension pneumocephalus is of extreme importance and a high degree of suspicion and expertise is required to reach an accurate diagnosis [3]. In this report, we describe a case of pneumocephalus caused by a ruptured cholesteatoma, which led to the sudden and unfortunate demise of the patient.

## Case presentation

**Figure 1:**
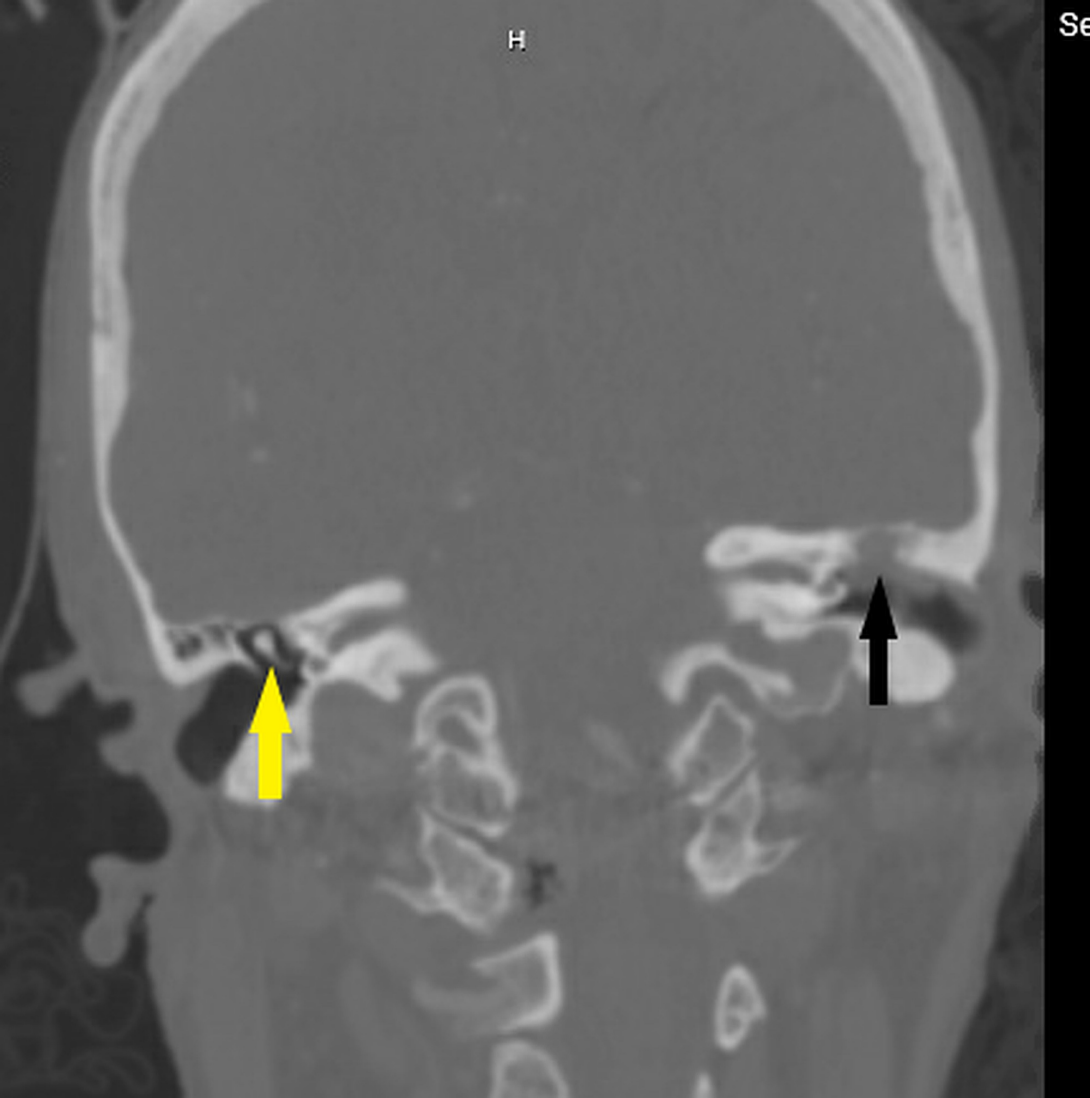
CTA of the patient The presence of cholesteatoma (black arrow) can be noted in the left middle ear cavity, visible as an opacification. Compare with normal-appearing malleus (yellow arrow) and the middle ear on the right side CTA: computed tomography angiography

A 64-year-old woman with a history of end-stage renal disease on hemodialysis and hypertension presented to the emergency department (ED) with acute-onset weakness and decreased mentation. On arrival to the ED, a code stroke was activated. Her National Institutes of Health (NIH) stroke scale score was calculated to be 30. She was noted to have a left-sided facial droop; pupils were pinpoint and showed a sluggish response to light and she had a positive Babinski response on the left. The Glasgow Coma Scale (GCS) score was 10/15. The vitals were as follows - blood pressure (BP): 180/102 mmHg; heart rate (HR): 98/minute; temperature: 97.1 °F; and oxygen saturation (SpO_2_): 100% on 3 L supplemental oxygen. Her labs showed a blood glucose of 146 mg/dl (normal level: <180 mg/dl), prothrombin time of 13.1 seconds (normal range: 11-13.5 seconds), and international normalized ratio (INR) of 1.1 (normal range: 1.1 or below). She had a white blood cell (WBC) count of 13.7 K (normal range: 4.5-11.0 K), hemoglobin of 13 g/dl (normal range: 12.1-15.1 g/dl in females), and a sodium value of 136 mmol/dl (normal range: 135-145 mmol/dl). Her creatinine was 2.0 mmol/L (normal range: 0.84 to 1.21 mmol/L) and glomerular filtration rate (GFR) was <15 ml/min (normal range: 90-120 ml/min), consistent with end-stage kidney disease. A CT scan and CT angiogram (CTA) of the head/neck was performed. The CT was unremarkable for any hemorrhage or ischemia but showed signs of free air in the cranial vault, consistent with pneumocephalus. The source became clear in the CTA as a cholesteatoma of the left middle ear cavity, which had resulted in the dehiscence of the tegmen tympani and scutum (Figure 1).

Neurosurgery was immediately notified of the findings. The patient’s respiratory status started to get worse and intubation was planned for her. A quick discussion was conducted with the family members who were present at the time, and it was decided to hold off on intubation. Comfort measures were called for, and the patient passed away in peace a few minutes later.

## Discussion

In 1884, Chiari reported the autopsy findings of pneumocephalus in a person who had suffered from ethmoiditis [4]. Later on, Dandy reported a case of otogenic pneumocephalus in 1926 [5]. Since then, cases have been documented more frequently in the literature. One-third of the cases of otogenic pneumocephalus result from traumatic events, one-third from otologic infections, one-third from procedures involving the mastoid cavity, and the remaining fall under the idiopathic category [2]. For pneumocephalus to occur, a fistulous connection is required between the cranial vault and the outside environment, which results in air migration [6]. The accumulation of air inside the cranium can be explained by the inverted “soda bottle” effect defined by Horowitz and the “ball-valve” mechanism proposed by Dandy respectively [7,8]. The cerebrospinal fluid leak from a defect creates a negative intracranial pressure in the former while a positive extracranial pressure forces air inside the cranial cavity in the latter with the intracranial contents acting as a flap valve to prevent the air escape [9,10]. The fistulous connection between a pneumatized temporal bone and the intracranial cavity is critical for the pathogenesis of spontaneous otogenic pneumocephalus (SOP), which is a rather rare entity with only 30 cases described so far [11]. Pneumocephalus secondary to an eroding cholesteatoma is also an uncommon finding, and that was seen in our patient.

**Figure 2:**
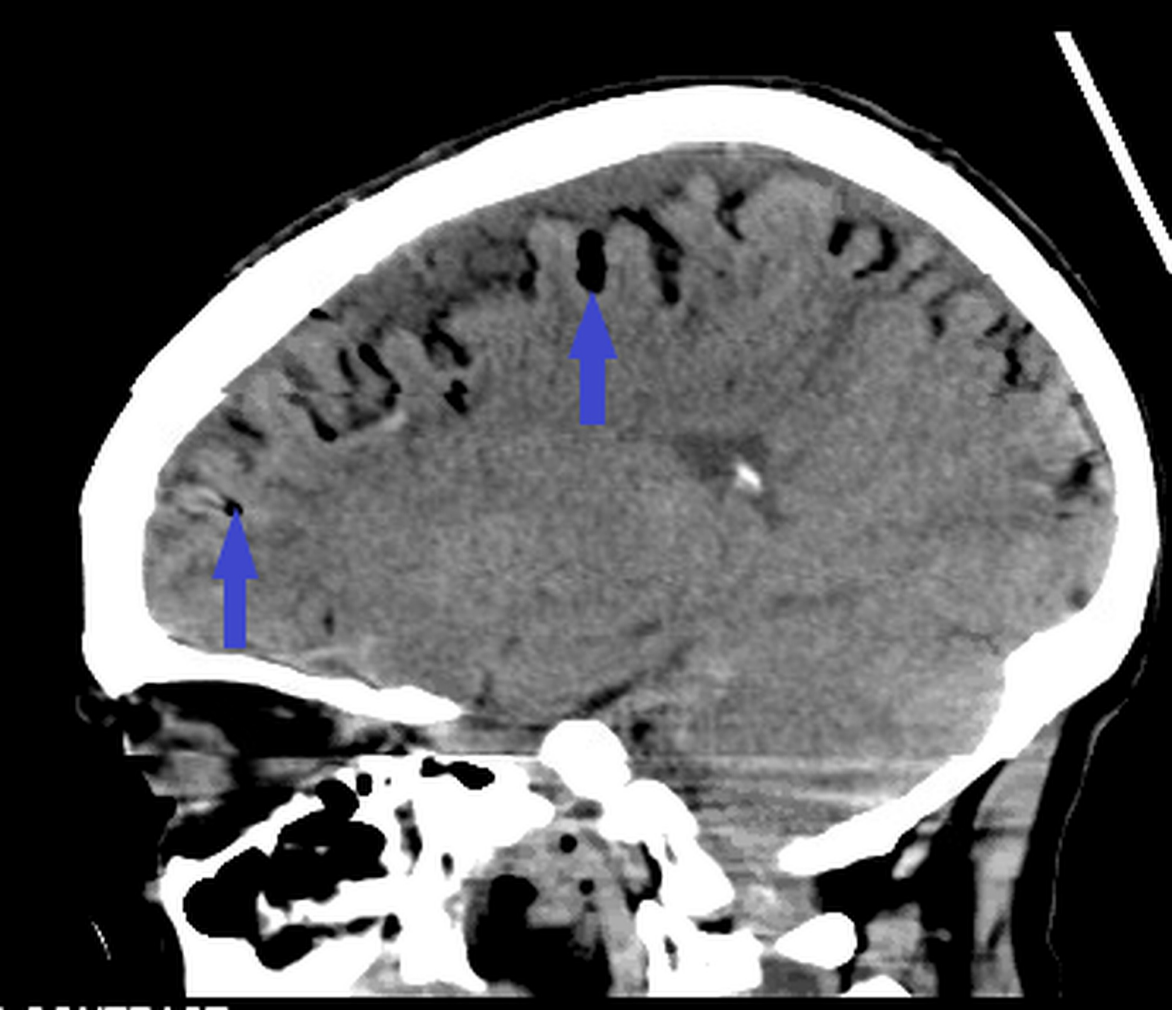
CT scan of the patient Note the presence of air bubbles (blue arrows) in the sulci diffusely (also known as the “air bubble” sign) CT: computed tomography

The diagnosis is usually accomplished with the aid of imaging. CT scan and MRI are the most commonly employed techniques, and they provide a fair degree of sensitivity and specificity [1,12]. Of clinical importance is the fact that the relatively benign pneumocephalus should be differentiated from tension pneumocephalus based on the characteristic imaging findings. The evidence of mass effect from the trapped air should be looked for earlier in the CT scan because it can result in herniation of the brainstem [3]. “Mount Fuji” sign, “peaking sign”, and “air bubble sign”, although not pathognomonic, are usually described by clinical radiologists when interpreting the CT scan findings [11-13]. The separation and compression of the frontal lobes secondary to the pressure of the trapped air as seen on the CT scan constitute the commonly reported “Mount Fuji” sign [3]. Both “Mount Fuji” and “air bubble” signs (presence of air bubbles diffusely) might herald the onset of tension pneumocephalus, but their prognostic value can be questioned [14]. The CT scan of our patient revealed the “air bubble” sign (Figure 2).

Although the majority of pneumocephalus cases are asymptomatic, the spectrum can range from headache and confusion in benign pneumocephalus to seizures and cardiac and respiratory arrest in tension pneumocephalus [12,15]. Spontaneous and secondary otogenic pneumocephalus can present with but is not limited to the symptoms of otalgia, aural fullness, otorrhea, and pain behind the mastoid [16]. Aphasia can develop after the onset of otologic symptoms and should alert the physician about the diagnosis. An increase in the middle ear pressure secondary to nose-blowing, Valsalva maneuver, or coughing can result in the development of the fistulous connection between an already pneumatized temporal bone and the cranial vault [8,10]. Symptoms can be masked if patients present with confusion and it is hard to get a good history as in our case.

Benign pneumocephalus, which is commonly seen after neurosurgical procedures, resolves with the resorption of the intracranial air. Fowler’s position is usually encouraged with the patient’s torso at an angle of 45-60 degrees in the bed; this specific position helps with breathing, resulting in improved oxygen delivery. Anti-pyretic medications and avoidance of Valsalva maneuvers have the added benefit of a faster recovery and, when implemented correctly, the measures have proven to be effective in more than 80% of the cases [12].

Studies have been conducted on the use of normobaric oxygen as a treatment. Different techniques can be utilized for the administration. A prospective study conducted in Germany found the use of normobaric hyperoxia at a 100% FiO_2_ delivered through the endotracheal tube to be an effective treatment strategy for pneumocephalus associated with posterior fossa surgeries; improved patient outcomes in terms of early recovery from anesthesia were also reported [17]. Hyperbaric oxygen results in better outcomes in terms of faster response and lesser side effects, as reported by Paiva et al. [18].

The treatment approach is primarily driven by the etiology of the disease process and the type of pneumocephalus. Tension pneumocephalus, regardless of the precipitating factor and the initial insult, is managed by decompression of the intracranial compartment, which can be achieved by drilling of burr holes, ventriculostomy, aspiration of the air, and craniotomy [3,15]. Evaluation for skull base fractures should be carried out in detail in post-traumatic cases as surgical repair is usually warranted [19]. Otogenic pneumocephalus can present secondary to any of the underlying middle ear and mastoid pathology or can be spontaneous as mentioned earlier. Temporal bone pneumatization has been correlated with the development of SOP [11]. With regard to the same concept, mastoid air cell volume has been found to be greatest in the third decade of life with a subsequent decrease with aging [8]. On the contrary, semicircular canal dehiscence is a direct correlate of bone demineralization, which is commonly found in older women [20]. CT scan is used to determine the mean skull base thickness (MSBT), which is linked to temporal bone pneumatization and is influenced by genetic factors [8]. It is an important tool, which, in conjunction with the MRI, can be used for the detailed evaluation of temporal bone anatomy, evidence of tegmen erosion, and intracerebral and epidural abscesses that need surgical drainage. Increased middle ear pressure from Eustachian tube dysfunction and repeated Valsalva maneuver can result in hyper-pneumatization of the temporal bone [11]. The repair of the primary defect usually results in the closure of the fistulous connection and seals the defect in the dura, which acts as the portal for air entry. Cartilage, free fascia, temporalis muscle graft, hydroxyapatite bone cement, and fibrin gel are used in different combinations; a multi-layered approach usually leads to the best outcomes [8,11]. These factors could have contributed indirectly to the causation of pneumocephalus in our case, in which the primary cause was an eroding cholesteatoma.

Antibiotics have a role in the treatment when meningitis and pneumocephalus are suspected concurrently and acute otitis media is diagnosed as the causative factor. A third-generation cephalosporin, either intravenous (IV) Rocephin (Roche AG, Basel, Switzerland) or cefotaxime in combination with IV vancomycin is the recommended therapy for otogenic meningitis with pneumocephalus [2]. The addition of steroids like dexamethasone results in improved neurological outcomes although their exact roles need to be more clearly defined.

It is of vital importance to keep in mind the causative factors, the knowledge of anatomy, and genetic variations in the architecture of the osseous floor of the middle cranial fossa as it is crucial for determining the most effective treatment approach for tension pneumocephalus.

## Conclusions

The relatively benign pneumocephalus is common after neurosurgical interventions and resolves spontaneously. However, it is advisable to maintain a low threshold for the diagnosis of the life-threatening tension pneumocephalus as the CT findings might not always corroborate. In addition, keeping a broad differential can often help in reaching an accurate diagnosis. An effective search for a middle ear pathology should be performed once an obvious cause like a traumatic insult has been excluded. Although less commonly reported, tension pneumocephalus secondary to an otogenic cause carries significant mortality and should be acted upon without delay. Further studies elucidating the causative factors and pathogenesis can influence our understanding of the pathology and can help us adopt life-saving preventive and treatment strategies.
